# The transformation of targeted killing and international order

**DOI:** 10.1080/13523260.2017.1336604

**Published:** 2017-08-01

**Authors:** Martin Senn, Jodok Troy

**Affiliations:** ^a^ Department of Political Science, University of Innsbruck, Innsbruck, Austria; ^b^ The Europe Center, Stanford University, Stanford, CA, USA

**Keywords:** Targeted killing, assassination, drones, international order, international law, morality, legitimacy

## Abstract

This article introduces the special issue’s question of whether and how the current transformation of targeted killing is transforming the global international order and provides the conceptual ground for the individual contributions to the special issue. It develops a two-dimensional concept of political order and introduces a theoretical framework that conceives the maintenance and transformation of international order as a dynamic interplay between its behavioral dimension in the form of violence and discursive processes and its institutional dimension in the form of ideas, norms, and rules. The article also conceptualizes targeted killing and introduces a typology of targeted-killing acts on the basis of their legal and moral legitimacy. Building on this conceptual groundwork, the article takes stock of the current transformation of targeted killing and summarizes the individual contributions to this special issue.

Targeted killing has been a perennial feature of human history. All along, members of political communities have slain their tyrannical rulers to establish just governance, have killed political leaders of other communities to enforce their interests, or have targeted enemy military leaders to turn the fortunes of war. Although targeted killing has persisted over the millennia in many different forms, its frequency and its moral, legal, and functional appraisal have varied significantly. In Ancient Greece, for example, a number of city-states passed legislation that allowed and rewarded the killing of tyrants to save democratic rule (Teergarden, 2014, pp. 5–8). In 15th- and 16th-century Europe, the killing of foreign leaders was both widespread and largely accepted as morally legitimate. In subsequent centuries, however, changing moral principles as well as the development of norms and laws at domestic and international levels restrained the use of targeted killing (W. Thomas, 2005). Although states (and non-state actors) continued to engage in targeted killings (Ford, 1985), they largely did so under the cloak of secrecy (Alston, 2010, p. 5).

In our day and age, targeted killing is undergoing yet another profound transformation. This current transformation of targeted killing has three dimensions. First of all, the group of states contemplating or using this type of violence has expanded and, in particular, the overall number of targeted-killing missions has increased significantly since the early 2000s and in the context of the escalating fight against terrorism. Second, the development and proliferation of drone technology (Horowitz & Fuhrman, 2015; Sayler, 2015) constitute a technological revolution for targeted killing that enables states to engage in the long-term surveillance and targeted killing of individuals with comparatively little risk. Finally, states that employ targeted killing have also started to abandon their policies of denial and to (hesitantly) engage in public justification (MacDonald, 2016). So targeted killing has moved from the fringes of undercover activity to the very core of policy-making in national security.

This transformation is unfolding against the background of ideas, norms, and rules that constitute the global international order. Existing scholarship on the relationship between targeted killing and international order predominantly focuses on the extent to which current practices of targeted killing comply with existing legal and moral ideas, norms, and rules, as well as on whether these principles should be adapted to meet the realities of armed conflicts in the 21st century (e.g. Blum & Heymann, 2010; Finkelstein, Ohlin, & Altman, 2012; Gunneflo, 2016; Melzer, 2008). This scholarship essentially analyzes the impact of the current international order on targeted killing and partially advocates changes to this order. Few scholars (e.g. Fisher, 2006; Fisk & Ramos, 2014; W. Thomas, 2005) have focused on the flipside of the relationship between targeted killing and international order, that is, on the question of *whether and how the current transformation of targeted killing is transforming the global international order.* So our knowledge of the impact that the use of targeted killing has on the ideas, norms, and rules of the global international order is still very limited. The articles in our special issue seek to fill this lacuna.

To get a handle on the central question of this special issue, we use a theoretical framework that conceives the interplay between violence and discursive processes as the behavioral dimension of an international order and ideas, norms, and rules as its institutional dimension. As students of order have long noted (e.g. Fukuyama, 2014, p. 583; Kalyvas, Shapiro, & Masoud, 2008, p. 1; North, Wallis, & Weingast, 2009, pp. 13–18), the management of violence is a key function of political orders as it delimits types of violence that violate or reinforce the principles of an order, that is, illegitimate and legitimate forms of violence (Hurd, 2017a). So violence contributes to the maintenance of an international order, yet, at the same time, it also leads to its transformation. In this maintenance and transformation of order through violence, discursive processes play an important role. Agents (de)legitimize the use of violence and (re)interpret the existing ideas, norms, and rules of an order in view of changing threats, technologies, and military doctrines.

Our introduction to this special issue proceeds in five steps. First, we review the existing literature to make a case for our work on the impact of targeted killing on international order. Second, we unfold the concept of international order and introduce our theoretical framework that conceives a dynamic, triangular interplay between violence, discursive processes, and the ideas, norms, and rules that make up an international order. Third, we shift the focus from international order to targeted killing as a type of violence. We conceptualize targeted killing and introduce a typology that distinguishes between four types of targeted-killing acts on the basis of their legal and moral legitimacy. Fourth, we inquire into the characteristics and facilitating conditions of the current transformation of targeted killing. Fifth and finally, we introduce the articles of this special issue by locating them in the context of our theoretical framework and briefly summarizing their arguments on how the transformation of targeted killing affects international order.

## The debate on the use, legitimacy, and impact of targeted killing

The current transformation of targeted killing has kindled considerable interest among scholars and has resulted in a fast-expanding literature. This literature consists of three branches that address the use, legitimacy, and broader impact of targeted killing.

The first branch on the use of targeted killing includes statistical studies on the frequency of targeting killing (Eisner, 2011; see also the descriptive statistics in Jones & Olken, 2009; McGovern, 2010), work on the history of targeted-killing strategies (F. L. Ford, 1985), and studies on how and why agents resort to this type of violence (David, 2003; Gazit & Brym, 2011; Iqbal & Zorn, 2008; Jacobsen & Kaplan, 2007; Plaw, 2008; Teergarden, 2014). In this context, scholars have also inquired into the trends and causes of the proliferation of drones as the preferred instruments of contemporary targeted-killing operations (Gilli & Gilli, 2016; Horowitz & Fuhrman, 2015; Sayler, 2015; see also critically Carvin, 2015). Another avenue of research in this first branch concerns the effectiveness of targeted killing. A number of scholars have argued that targeted-killing or decapitation strikes are effective in the sense that they undermine the operational capabilities and resolve of enemy agents (Byman, 2006; Johnston, 2012; Price, 2012; Tiernay, 2015) or, at least, the moral within the attacking country (Wey, 2015). This argument of strategic effectiveness has been challenged by scholars who argue that the targeted killing of state (Hosmer, 2001; Pape, 1996) and non-state agents (Gill, Piazza, & Horgan, 2016; Hafez & Hatfield, 2006; Jordan, 2009, 2014; Kaplan, Mintz, Mishal, & Samban, 2005) has no effects or even negative effects such as increasing enemy resolve or inciting retributive attacks (see also Carvin, 2012, and, more recently, Abrahams & Mierau, 2015; Johnston & Sarbahi, 2016; Lehrke & Schomaker, 2016; Morehouse, 2015). While these studies address the effectiveness of targeted killing in armed conflict, Bob and Erikson Nepstad (2007) analyze factors that influence the effects of killing leaders in social movements.

The second and largest branch discusses the legitimacy of targeted killing, that is, whether acts of targeted killing comply with legal and moral principles, and ponders whether states should amend these principles to meet the realities of armed conflicts. In this context, a fundamental question is whether the paradigm of law enforcement, which is restrictive regarding the use of lethal force against individuals, or the more permissive paradigm of armed conflict governs state-mandated targeted killing (Blum & Heymann, 2010; Falk, 2014; Gross, 2006; Melzer, 2008; Otto, 2012; see also part II in Finkelstein et al., 2012). Scholars have tried to come to terms with the fact that targeted killing sits uneasily between these two paradigms by suggesting the integration of their elements (Farer & Bernard, 2016; Kretzmer, 2005), the reinforcement of their distinction (Meyer, 2012), or the abandonment of the paradigms in favor of alternative frameworks for judging the legality of targeted killing (Hakimi, 2012). In addition, scholars have also debated whether self-defense is a viable justification for targeted killing (Corn, 2012; Guiora, 2004; Martin, 2012; Schmitt, 1992, 2010; see also Brincat, 2008a, 2008b, 2009, for a discussion of self-defense in the case of tyrannicide) and have inquired into the legality of using (targeted) force as self-defense against states that are “unwilling or unable” to prevent non-state actors from using their territory as a base for hostilities against other states (Ahmed, 2013; Deeks, 2012; Reinold, 2011a, 2011b; Williams, 2013).

Focusing specifically on the paradigm of armed conflict, which the United States and Israel have invoked to justify their killing of terrorists actors, scholars have debated whether the *jus ad bellum* and *jus in bello* principles of Just War Theory still apply in asymmetric conflicts between states and terrorist organizations, or should be supplemented with new principles of a *jus ad vim* (Walzer, 2006)*.* This *jus ad vim* would consist of principles governing the use of force short of war, which would include targeted killing (Brunstetter & Braun, 2011; S. B. Ford, 2013; Frowe, 2016). Focusing on *jus ad bellum*, Altman and Wellman (2008) and Statman (2004, 2012) argue that once large-scale military intervention becomes morally permissible, so does targeted killing. Aloyo (2013) takes this reasoning one step further by speaking of a moral obligation to choose targeted killing over other alternative measures that entail more harm to innocents (see also Strawser 2010 for a similar argument on the use of drones).

The second branch also addresses aspects of *jus in bello* and, in particular, the question of discrimination and proportionality in targeted-killing missions. As for the former, scholars have grappled with the issue of how to discriminate terrorists from civilians and have formulated a number of proposals. These proposals include the identification of legitimate targets on the basis of individual conduct (Guiora, 2012, 2013), organizational affiliation (Gross, 2010, pp. 108–120), or a combination of both principles (Maxwell, 2012; Ohlin, 2012). Closely related to this debate about discrimination is the debate about the proportionality of targeted killings. Participants to this debate have supported (Etzioni, 2013; McNeal, 2012; Plaw, 2013) or challenged (Braun & Brunstetter, 2013; Crawford, 2013; O’Connell, 2010) the view that (the use of drones in) targeted killing minimizes civilian casualties in conflicts. Finally, scholars have also debated whether the (assumed) *in bello* proportionality of drones affects the propensity of states to use force, that is, whether drones lower the threshold for the use of force (Anderson, 2012; Brunstetter & Braun, 2011; Kreps & Kaag, 2012; Ohlin, forthcoming; Strawser, 2014).

The transparency and accountability of targeted killing is another dimension of legitimacy. Scholars have criticized the lack of domestic accountability of targeted-killing strikes by the United States (Alston, 2011; Foust, 2011; Singer, 2012) or rejected this criticism by pointing to the effectiveness of a complex network of mechanisms that ensure the accountability in U.S. targeted-killing missions (McNeil, 2014). Other scholars propose measures on how to increase transparency on both domestic (Guiora, 2013; Johnson, 2007; Murphy & Radsan, 2009) and international levels (Buchanan & Keohane, 2015). Finally, a number of studies have moved beyond assessing the legitimacy of targeted killing by analyzing the legal and moral arguments (Brunstetter & Jiminez-Bacardi, 2015; MacDonald, 2016) as well as the terminology (Waldron, 2016) that state and non-state actors use in (de)legitimizing targeted killings.^1^

The third and final branch of the literature inquires into the broader impact of targeted killing. A first set of scholars focuses on the impact that the targeted killing of state leaders has on domestic political orders (Iqbal & Zorn, 2008; Jones & Olken, 2009). A second set addresses targeted killing in the context of contemporary conflicts and debates local and regional effects such as increasing anti-American sentiments and psychological distress of local populations or the destabilization of governments and escalation of regional conflicts (Boyle, 2013; Fair, Kaltenthaler, & Miller, 2014; Farhat, 2010; Hudson, Owen, & Flannes, 2011). Only few scholars have extended the scope further by addressing the effects of targeted killing on the current international order. Ward Thomas (2001, 2005) focuses on the anti-assassination norm that has consolidated together with the Westphalian international order and argues that the rise in targeted killing, or what he calls “the new age of assassination” (W. Thomas, 2005), has resulted in a decline of this norm. While Ward Thomas addresses normative decline, Fisher (2006) concludes in his work that a “norm permitting the use of targeted killing for counter-terrorism purposes appears *likely to emerge* [emphasis added] and spread successfully” (p. 757; see also Jose, 2016; Lantis, 2016). In a similar vein, Fisk and Ramos (2014, 2016) argue that a norm of preventive self-defense slowly supersedes the existing prohibition of the preventive use of force (see also Boyle, 2015, pp. 119–121; Martin, 2012, p. 248ff.). Although these studies point to the relevance of inquiring into this dimension of the nexus between targeted killing and international order and provide a number of relevant insights, our overall knowledge of whether and how the current transformation of targeted killing is affecting the ideas, norms, and rules that constitute the global international order is still very limited. This special issue seeks to advance our understanding of this important question as well as to stimulate a sustained scholarly debate on it.

## A conceptualization of international order and its transformation

A systematic inquiry into the impact of targeted killing on international order presupposes a clear understanding of the essential properties of both phenomena; that is, it requires us to conceptualize both international order and targeted killing. In our laying of this conceptual groundwork for the special issue, we build on Goertz’s (2006) distinction between three levels of a concept in the social sciences. According to Goertz (2006, p. 30), the *basic level* of a concept includes a noun, which we use to refer to a phenomenon, a broad and very abstract understanding of the phenomenon, its opposite (or negative pole), and the relationship between the phenomenon and its opposite (e.g. between war and peace). The *secondary level* features the constitutive elements of a concept or the characteristics that make up the basic-level concept. Finally, the *data level* consists of a set of indicators that link the concept to empirical data (Goertz, 2006, pp. 6–7). In this section, we will introduce the basic and secondary-level concepts of international order and then present the basic and secondary-level concepts of targeted killing, as well as an associated typology of targeted-killing acts in the subsequent section.

A conceptualization of international order necessarily unfolds in two steps: It first illuminates the phenomenon of order in the social or political realm and then addresses the attribute of the “international,” that is, the peculiarities of an *international* order (see also Lascurettes, 2011; Tang, 2016). As Wrong (1994, 41) notes in his treatise on *The Problem of Order*, “order” denotes both regularity and rule (see also Anter, 2007; Elster, 1989, p. 1; Hurrell, 2007, p. 2). In other terms, we can distinguish between two interrelated but analytically distinct basic-level concepts or dimensions of order. This distinction is also apparent in our everyday use of the word: While “there is order” refers to a regularity such as a patterned movement of vehicles over a crossroad, “there is *an* order” denotes the existence of a rule or institution such as a traffic regulation that enables regularity in the behavior of agents.

In the case of order as regularity, that is, the behavioral dimension of order, the relationship between “order” and the negative pole of “disorder” is continuous. So “social order is a matter of degree. Order is never so fully present in concrete social reality as to exclude all deviations, unpredictabilities, mistaken perceptions, and accidents” (Wrong, 1994, pp. 9–10). In other terms, there is always wiggle room for agency that produces disorderly behavior. Likewise, in the case of order as rule, that is, the institutional dimension of order, the “presence of an order” and the “absence of an order” mark the positive and negative poles of a continuum. Between the two poles of a stable and uncontested order and a complete breakdown or absence of an order is the “gray zone” (Goertz, 2006, p. 34) of partial contestation and transformation. Lastly, the relationship between the two basic-level concepts or dimensions of order is dialectical (Anter, 2007, p. 58). On the one hand, the institutional dimension allows for the behavioral regularities that reproduce the institutional dimension. On the other hand, there is room for agency that results in irregular, disorderly behavior that challenges and may ultimately transform the institutional dimension.

Moving on to the secondary level, we propose two definitions of order. First, we define the behavioral dimension of order as a *relatively stable and thus recognizable and predictable pattern of agents’ behavior or its outcomes*. The notion of order as regularity implies that the behavior of agents and the outcomes of their interactions are relatively stable over time (Tang, 2016, pp. 33–35). As we already mentioned above, the attribute of “relative” stability denotes that regularity in the social realm is never perfect, but usually coexists with varying degrees of accidental, creative, and transformative irregularity. Eventually, this regularity of behavior and outcomes enables agents to recognize a social system as orderly and thus to form expectations about the future (Hayek, 1968, p. 11, 1973, p. 36; Tang, 2016, p. 35).

Second, we define the institutional dimension of order as *an evolved or designed institution or set of institutions that emerges from the needs of agents within a social system*. At the core of this secondary-level concept is the notion that an order is constituted by two elements: an institution that shapes the behavior of agents, that is, an “ordering mechanism” (Mazarr, Priebe, Radin, & Stuth Cevallos, 2016, p. 8) or “ordering principle” (Lorand, 1994, p. 307), and a “need” (Hoffman, 1987, p. 85) or ordering imperative that drives the creation and maintenance of an order. The institutions or ordering mechanisms can take on a variety of forms and degrees of formality such as intersubjective ideas, implicit or explicit norms, and rules. Moreover, they can evolve over time as a by-product of interaction or be the result of a conscious and purposive design by actors.^2^ The need for ordering imperative that drives the formation and maintenance of an order can range from common needs such as individual survival, to collectively agreed needs within a group of agents such as the need to stop the proliferation of nuclear weapons. Finally, an order exists and evolves within a social system. Together with the agents and their interactions, it is a constitutive element of social systems (Gilpin, 1981, pp. 26–27).^3^

Building on the distinction between two basic-level concepts of social order and the associated secondary-level concepts, we are now in a position to conceptualize “international order” as a relatively stable pattern of behavior or outcomes among states as principal agents and “an international order” as a set of imperatives and institutions that give rise to this discernible regularity.^4^ Past and present international orders have varied in their degree of behavioral regularity and stability, the nature of their institutional foundations, and their scope; that is, they have emerged in different issue areas such as the use of nuclear energy (Walker, 2011) and have featured in varying numbers of states. While some international orders have been limited to a smaller number of states (or state-like polities), such as past and present regional orders in Europe or East Asia (Ringmar, 2012; Suzuki, Quirk, & Zhang, 2014), others have been global in their scope.

In this special issue, we focus on the *global international order* or the so-called Westphalian international order as a set of institutions that constitute the state as the principal agent and fundamental rules that shape interaction between states.^5^ Developing in 16th- and 17th-century Europe (Holsti, 2004, pp. 121–128; Johnson, 2014) and gradually expanding on a global scale, this order builds on the fundamental principle or bedrock institution of sovereignty.^6^ As Holsti (2004) notes in this context, “[s]overeignity is a *constitutive* rule of statehood because it defines and helps create legitimate agents, those who have a unique juridical personality” (p. 114). So the institution of sovereignty constitutes the state as the principal legitimate agent and defines it as a polity that possesses supreme and effective authority with a demarcated territory, is not subject to external authority, and is recognized as a state by other states (Holsti, 2004, pp. 113–114; Krasner, 2001, p. 18, 2013). In addition, sovereignty also has a regulative dimension in that it creates rights and obligations for states such as diplomatic representation and noninterference (Holsti, 2004, p. 113, 117) and has triggered the development of a plethora of further institutions that regulate the interaction of states.

A central dimension of the institutional development within the Westphalian order has pertained to the use of violence between states.^7^ This development of institutions that prohibit and allow certain forms and means of violence between states has followed from two imperatives or needs: to ensure the long-term stability of states and a state-based order and to avoid unnecessary harm to human beings and infrastructures in times of war. The emergence and consolidation of the institution of state sovereignty were already accompanied by the development of a norm prohibiting the targeted killing of state leaders (see also Großklaus, 2017). The rise of this norm followed not only from growing moral revulsion against treacherous killing (e.g. by poisoning), but also from concerns about instability and disorder that would follow from the killing of state leaders (W. Thomas, 2001, 2005). More recently, the United Nations (1973) and the Charter of the Organization of African Unity (1963) explicitly prohibit the killing of high-level state representatives.^8^

The management of violence in the Westphalian order has also progressively extended into the domain of warfare. While the Just War doctrine had long provided a moral framework that had influenced state justifications of war (Lesaffer, 2015), the 19th and 20th centuries witnessed the development of a complex institutional network governing the conduct of war and the recourse to war. States first formulated rules on the conduct of war (*jus in bello*) in a series of conferences and conventions (e.g. the Hague and Geneva Conventions). In this body of so-called international humanitarian law, states seek to strike a balance between “the need to be able to fight effectively on the battlefield and [the] desire to avoid unnecessary harm to combatants and the civilian population” (Schmitt, 2011, p. 89). This body of law rests on a number of fundamental principles (Crawford & Pert, 2015, pp. 41–48) such as the *distinction* between noncombatants (e.g. civilians, medical units, or wounded soldiers) and combatants, the associated principle of *discrimination* that violence must not be directed against noncombatants, the principle of *proportionality* in the sense that the military benefits of the use of force must outweigh the damage to civilian populations and objects, and the principle of *unnecessary suffering* that seek to constrain means and methods of war which cause superfluous injury or unnecessary suffering (e.g. shrapnel or incendiary weapons).

In addition to rules on the conduct of war, the current international order also features rules on the resort to war (*jus ad bellum*). After attempts to regulate war in the Covenant of the League of Nations (1919) and to prohibit offensive war in the Kellogg–Briand Pact of 1928, the Charter of the United Nations now includes a comprehensive prohibition of “the threat or use of force” in Article 2(4). The only (relevant) exceptions to this prohibition are acts of self-defense (Art. 51), the use of force authorized by the Security Council of the United Nations (Art. 42), and anticipatory or preemptive self-defense, which is recognized in international customary law.^9^

**Figure 1.**
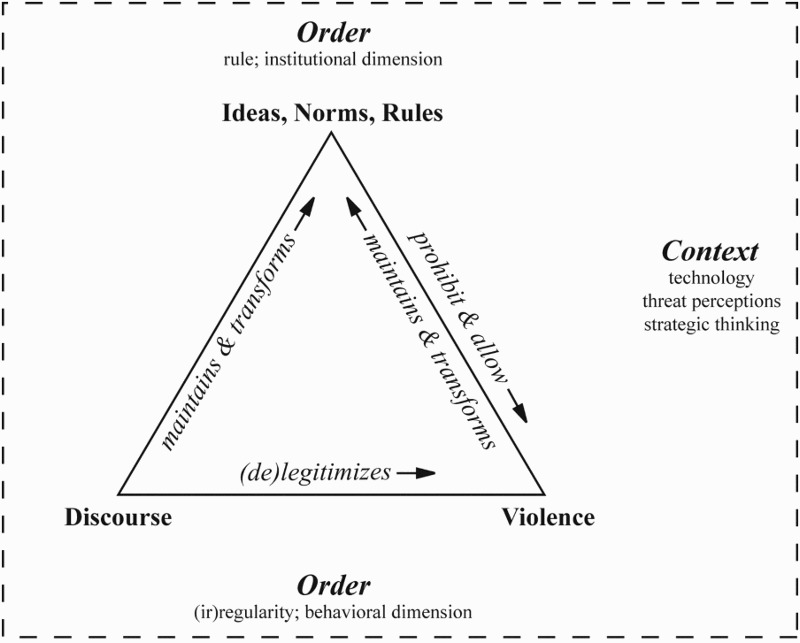
The maintenance and transformation of international order.

As the preceding paragraphs already suggest, the institutional dimension of the Westphalian order is by no means static, but has undergone a myriad of transformations. Focusing on the nexus between international order and violence, we conceive the maintenance and transformation of an international order as a triangular process (see Figure 1). The institutional dimension of an international order contains ideas, norms, and rules that prohibit and allow certain types of violence; that is, the institutional dimension manages violence by delimiting legitimate and illegitimate types (Hurd, 2017a). The use of violence may contribute not only to the maintenance of an international order, but also to its transformation. So, on the one hand, violence may reinforce an international order by punishing behavior that violates its principles, as in the case of military operation against Saddam Hussein’s Iraq to reinstate the sovereignty of Kuwait. On the other hand, the use of violence may also transform the ideas, rules, and norms of an international order. For example, scholars have argued that current practices of using force preventively, that is, against emerging rather than imminent threats, are fueling the emergence of a norm of preventive self-defense (e.g. Fisk & Ramos, 2014; Nichols, 2016).

Yet violence rarely operates alone in the maintenance and transformation of order. It is accompanied by discursive processes in which agents create and communicate meaning.^10^ As Figure 1 illustrates, discursive processes operate in two directions. First, agents argue over the extent to which a potential or actual use of violence is in line with the existing principles of an order (Hurd, 2017a; Morrow, 2014, p. 32). In other terms, they legitimize or delegitimize certain forms and instances of violence. For example, coalitions of state and non-state actors have successfully delegitimized the use of landmines and cluster munitions, and are currently engaged in the delegitimization of nuclear weapons by emphasizing their indiscriminate nature and the grave humanitarian consequences of their use (Borrie, 2014). Second, agents also (re)interpret the ideas, norms, and rules that make up the institutional dimension of an order and thus also contribute to its change. A recent example is the reinterpretation of “imminence” in the course of the ongoing fight against transnational terrorism and the proliferation of weapons of mass destruction. Decision-makers in the United States and the United Kingdom have argued that the traditional criteria of imminence as they were stipulated in the Caroline Case^11^ have to be expanded in view of terrorist actors and states that clandestinely develop weapons of mass destruction (Arend, 2003; Watson, 2017).

What accounts for this transformative agency or, in other terms, which factors lead actors to challenge and change the institutions that govern the use of violence? On the one hand, and as recent scholarship on the contestation of norms has emphasized (Sandholtz, 2008; Wiener, 2014), the indeterminacy of ideas, norms, and rules as well as conflicts between these elements of an international order are endogenous factors that trigger transformative agency (Morrow, 2014, pp. 32–33). On the other hand, changes in military technologies, threat perceptions, and strategic thinking are exogenous, contextual factors that stimulate new types of violence, discourse, and thus ultimately the transformation of international order. Now that we have discussed the notion of (international) order and have thus built the first conceptual pillar of our special issue, we will establish its second pillar by focusing on targeted killing as a form of violence in the next section.

## A conceptualization of targeted killing

The academic debate about targeted killing has featured a plethora of labels such as “summary execution,” “extrajudicial killing,” “named killing,” “targeted elimination,” “targeted killing,” or “(state-sponsored) assassination,” and their respective definitions. In particular, this debate has interacted with long-established and closely related arguments about assassination (Bell, 1972; F. L. Ford, 1985; Zellner, 1974). As a consequence, scholars have used the labels of “assassination” and “targeted killing” interchangeably (e.g. Altman & Wellman, 2008), have treated targeted killing as a special type of assassination (e.g. Tesón, 2012) and vice versa (e.g. Himes, 2016), or have argued over categorical differences between targeted killing and assassination (e.g. Patterson & Casale, 2005; Wachtel, 2005). As the debate about the distinction between assassination and targeted killing as illegal and legal forms of violence demonstrates, scholars have frequently included legal and moral judgments in their labels and definitions of targeted killing (Knoepfler, 2010, pp. 467–468).

Our conceptualization starts from the premise that concepts in the social sciences should be as nonnormative or value-free as possible (Carter, 2015; Dowding, 2016, pp. 194–195). In other terms, concepts should exclude, to the extent possible, evaluative elements (Carter, 2015, p. 280).^12^ It is therefore important to distinguish between the concept that delimits the essential characteristics of targeted killing as a form of violence and the discourse about the legitimacy of targeted-killing acts within different moral and legal contexts (Ben-Yehuda, 1990; Knoepfler, 2010). As Ben-Yehuda (1990) succinctly puts it,

[t]he point is that there is no “real” or “objective” meaning of an event because meaning is negotiable and culturally dependent. What for one person or (group) is a (bona fide) political assassination may be interpreted as a simple murder for another person (or group). (p. 334)

Following this fundamental distinction, we first introduce a concept of targeted killing and then develop a typology of targeted-killing acts on the basis of their moral and legal legitimacy.

At the basic level, we retain the label of “targeted killing” to denote a form of lethal violence directed against preselected individuals. Between targeted killing and its conceptual opposite of un-targeted or random killing lies a continuum of killings with different degrees of targetedness, such as mass-casualty terrorism, carpet bombings, or cruise-missile strikes. At the secondary level, we build on Melzer (2008) and define targeted killing as *the use of intentionally lethal violence against a prominent or culpable person or a small group of persons* (the target) *not in the physical custody of the agent using violence* (the source). This definition includes a number of constitutive elements, which we have to discuss in further detail to delimit targeted killing from other types of violence.

First of all, targeted killing obviously involves lethal violence. Although the current debate about targeted killing puts an emphasis on the use of unmanned aerial vehicles (or drones), targeted killing involves a large number of methods (Alston, 2010, p. 4) such as shooting at close or long ranges, stabbing, suffocation, bomb attacks, and contamination with toxic or radioactive substances. Second, the use of lethal violence is intentional (Aloyo, 2013, p. 349; Altman, 2012; Knoepfler, 2010, p. 470) in the sense that the source has a preconceived plan to kill a human being or group of human beings. This criterion of intentionality sets targeted killing apart from unintentional killing such as “rage-induced or reflex-controlled … killing” (Protevi, 2008, p. 409) or the incidental or accidental killing of noncombatants in warfare, that is, collateral damage (Coady, 2013).

Third, and most importantly, the use of lethal violence is directed against a person or group of persons that the source considers prominent or culpable. These two attributes of the target denote that targeted killing involves a process of selecting individuals due to their elevated positions in religious, political, or military hierarchies,^13^ or the appraisal that their behavior has violated (or will violate) a community’s legal or ethical principles. One of these attributes has to be present for a homicide to be targeted killing, but they can (and often will) also be present in tandem, as, for example, in the case of tyrannicide (Brincat, 2008a, pp. 213–214; George, 1988, p. 402). This attribute-based selection of individuals delimits targeted killing from anonymous killing as it occurs in war, mass-casualty terrorism, or rampages. It also delimits targeted killing as a form of violence in the public sphere from killings that happen in the private sphere. In the case of targeted killing, the source uses lethal violence against a position in a hierarchy rather than the person holding the position, or it uses violence to prevent or sanction a public wrong committed by a person. So the killing of a political leader in a personal feud or mugging would not qualify as targeted killing. Fourth, and finally, targeted killing pertains to killing individuals or groups that are not in the physical custody of the actors using violence. As Melzer (2008) notes, targeted killing is “extra-custodial killing” that differs from execution as killing in custody (p. 4).

Unlike alternative definitions, this definition does not add and unpack further attributes of the source, target, or context in which targeted killing takes place. As for the source, a number of recent studies include the criterion of state sponsorship (e.g. Altman, 2012, p. 5; David, 2003, p. 112; de Wijze, 2009, p. 308). Yet this narrow focus on state agency smuggles claims about the legitimacy of targeted killing into its definition. It implies the state as having, in Max Weber’s (1992) sense, “the monopoly of legitimate use of physical force [translated by the authors]” (p. 6). However, throughout human history, both non-state actors in the form of single individuals, small groups, or broader movements, and state actors have engaged in *acts* of targeted killing (Eisner, 2011; Ford, 1985; Jones & Olken, 2009), irrespective of whether this use of force was deemed legitimate or not. Neither does our definition specify whether the target is a state representative or a non-state actor, or where the act of killing takes place, that is, in which territorial and legal context.

Although the characteristics of the source, target, and (legal and moral) context are not relevant for the concept of targeted killing per se, they are important for making judgments about the legitimacy of specific instances of targeted killing (Table 1). These judgments about the legitimacy of targeted killings provide the basis for our typology that we now introduce as a second step in our conceptual discussion of targeted killing. More precisely, this fourfold typology follows from judgments about the “legal legitimacy” and “moral legitimacy” of targeted killings, that is, judgments about the degree to which these instances are in line with relevant legal and moral principles in a given context.^14^

As Table 1 indicates, type I within our typology subsumes targeted killings that actors judge to have both legal and moral legitimacy. For example, current international law permits the targeted killing of a high-ranking military commander in the context of an armed conflict as long as it does not involve the use of treacherous means.^15^ In addition, the dominating view in contemporary Just War Theory, or what Haque (2017) calls the conventionalist view, holds that “combatants on all sides enjoy symmetrical moral permissions” in the sense that “[a]ll combatants are morally permitted to intentionally harm opposing combatants and to inflict necessary and proportionate collateral harm on noncombatants” (p. 19). So the killing of a military commander or terrorist leader in the course of hostilities would be both legally and morally legitimate.

**Table 1.** A legitimacy-based typology of targeted-killing acts.

Type II refers to targeted killings that actors consider to have moral legitimacy but lack legal legitimacy. A case in point is the targeted killing of tyrants, or tyrannicide. As Brincat (2009) notes in his lucid analysis, tyrannicide is currently prohibited by domestic laws and by at least three strings of regulations in international law: the prohibition against treacherous killing as codified in the Hague and Geneva Conventions, the New York Convention on internationally protected persons, and the prohibition of the use of force in international relations. At the same time, there is a long history of reasoning about the moral legitimacy of killing tyrants for the good of the community under their rule (Esquivel, 1979). Tyrannicide may also appear morally legitimate as a form of humanitarian intervention, that is, to put an end to large-scale violations of human rights. As Beres (1990) notes in this context: “If, after all, the assassination of a Hitler or a Pol Pot could have saved thousands of even millions of innocent people from torture or murder—it would be a far greater crime not to attempt such an assassination than to actually carry it out” (p. 857).^16^

Targeted killings that actors judge to be legally legitimate but at the same time morally illegitimate make up type III. For example, the so-called revisionist view of Just War Theory argues against the moral equality of combatants (McMahan, 2009). Haque (2017) pointedly summarizes this argument as follows:

Combatants who fight for an unjust cause (such as territorial conquest) typically pose unjust threats to opposing combatants and thereby lose their moral right not to be killed. In contrast, combatants who fight for a just cause (such as national self-defense) typically pose just threats to opposing combatants and therefore retain their right not to be killed. (p. 22)

So in this understanding of Just War Theory, the targeted killing of a high-ranking combatant may be in line with the legal principles that govern the use of violence but at the same lack moral legitimacy if this combatant is fighting for a just cause and the attacker is fighting for an unjust cause.^17^

Finally, type IV covers targeted killings that actors deem as lacking both legal and moral legitimacy. The killing of a head of state in a functioning democracy during peacetime (such as President Kennedy) would be a case in point. This type of killing is prohibited under domestic and international law (see type II) and cannot be reasonably justified by moral principles. As Walzer (2013) notes, “[i]n democracies, [political assassinations] can never be justified.”

This fourfold typology of targeted killing moves beyond the diffuse dichotomy between (legal) targeted killings and (illegal) assassinations^18^ by enabling a more fine-grained mapping of judgments about different forms and specific instances of targeted killing. In addition, the typology also allows us to trace the changes in the legal and moral appraisal of different forms of targeted killing and the changes in the underlying legal and moral principles. For example, while tyrannicide was a targeted killing of type I in Ancient Greece, it currently sits between types II and IV. In a similar way, the targeted killing of high-ranking state representatives was between types II and I in the 15th- and 16th-century Europe and is now a case of type IV. The articles in this special issue focus on state-sponsored targeted killing of terrorist actors as yet another form of targeted killing and seek to uncover whether its current use is having an impact on the ideas, norms, and rules that make up the current international order.

## The current transformation of targeted killing

As we noted at the beginning of this article, targeted killing has always been a feature of human societies, but its frequency and its moral, legal, and functional appraisal have varied over the millennia. In the history of targeted killing, the consolidation of the Westphalian order and its fundamental institution of state sovereignty marked a watershed moment. Driven by growing moral revulsion against the slaying of state leaders and concerns about political instability that would follow these acts, a norm stigmatizing the targeted killing of state leaders developed as an element of the emerging order of sovereign states. What had been a widespread and legitimate practice came to be regarded as uncivilized and perilous (W. Thomas, 2001, 2005). In spite of the norm, states have occasionally attempted to kill foreign (state and non-state) leaders, but they have tried hard to conceal their actions and faced international resistance once they were disclosed (Alston, 2010, p. 5).

Since the turn of the millennium, targeted killing has undergone yet another profound transformation, which comprises three dimensions. First, the number of targeted-killing missions against non-state, terrorist actors has increased significantly (see Table A1 in the appendix). The United States has emerged as the leading state in the use of targeted-killing tactics, in particular during the Obama administration, but also Israel, the United Kingdom, Russia, Iraq, Pakistan, and Nigeria have resorted to targeted killing in recent years (Ackerman, 2015; Alston, 2010; Burgers & Romaniuk, 2016; Hennigan, 2016).

Second, the rapid development and proliferation of surveillance and drone technology constitute a technological revolution in targeted killing. Drones can monitor suspects for extended periods of time and deliver deadly attacks with comparatively little (immediate) risks for the operator. This capacity for long-range surveillance and strikes without boots on the ground is particularly appealing for democratic regimes as they are under more pressure to lower the costs of military intervention. Yet recent research also suggests (Horowitz & Fuhrman, 2015, pp. 34–40) that drone technology is equally appealing for autocratic regimes which can use it for the control and repression of domestic opposition. At the moment, “[n]early 30 countries … have armed drone programs,” while almost as much countries “possess advanced unarmed drones” (Horowitz & Fuhrman, 2015, p. 1).^19^ In addition, drone exports by Israel, the United States, and, more recently, also by China will put more states within reach of a capacity for targeted-killing operations.

Third, states are slowly but steadily abandoning their policies of secrecy and denial with regard to targeted killing. Israel was the first to acknowledge the targeted killing of a terrorist actor in November 2000 and the United States followed suit by acknowledging their first drone strike outside an official warzone in 2002 (Brunstetter & Jiminez-Bacardi, 2015, pp. 181–182). Ever since, state representatives have repeatedly justified them on both legal and moral grounds (MacDonald, 2016), even though many details of targeted-killing operations are still closely guarded (Savage & Shane, 2016).

What accounts for this transformation of targeted killing? In what follows, we seek to identify a number of facilitating conditions. Given the purpose and constraints of this article, we can only offer a set of plausible hypotheses without engaging in a detailed investigation of them. A first and central factor accountable for the transformation of targeted killing is the change in threat perceptions that has taken place roughly since the end of the East–West Conflict. While concerns over terrorist activities had already been present for decades, they have become more prominent since the end of the bloc confrontation and with a series of large-scale attacks in the 1990s and the 2000s. States see themselves confronted with highly dangerous non-state adversaries that use remote and often poorly governed or violence-stricken areas to recruit and train new members as well as to prepare attacks. This shift in threat perceptions has been accompanied by new ideas on how to effectively cope with new threats. The paradigm of “effect-based operations” (Dill, 2015, p. 93) envisages enemy actors as networks and builds on the assumption that the elimination of central nodes will lead to a substantial weakening or collapse of the network. In the context of this paradigm, targeted killing appears as an effective and attractive solution (see also Haas & Fischer, 2017).

Beyond changes in threat perceptions and military thinking, the surge of targeted killing has been facilitated by an ongoing paradigm shift in international law. As Ruti Teitel (2011) argues in this context, “[t]he normative foundations of the international legal order have shifted from an emphasis on state security—that is, security as defined by borders, statehood, territory, and so on—to a focus on human security: the security of persons and peoples” (p. 4). This shift has prepared the ground for targeted killings in at least three ways. First, the paradigm of human security not only fosters the protection of individuals, but also entails a shift toward individual accountability (Ainley, 2008; Sikkink, 2011; W. Thomas, 2005). So the individual perpetrator, the actor rather than the act, has become the target of retributive actions by third parties. Individuals are held responsible for political actions that inflict harm on others and are eventually brought to justice.

Second, this ongoing paradigm shift in international law is intertwined with an ongoing paradigm shift in international norms. For example, the debate about the notion of a Responsibility to Protect, which developed as a part of the paradigm change toward human security (Kaldor, 2007) and the global promotion of a unified Human Rights regime (Hopgood, 2013), has provided significant impetus (or established argumentative patterns) for public justifications of targeted killings (see also W. Thomas, 2005, pp. 30–31). So, if an armed intervention is principally deemed morally permissible (out of responsibility), it “becomes untenable to hold that political assassination [i.e. targeted killing] is impermissible in principle” (Altman & Wellman, 2008, p. 228; de Wijze, 2009). For several states that are among the core contributors to the debate about the Responsibility to Protect, this concept has solidified a particular normative understanding of the notion of their own sovereignty. In particular, Western States tend to understand sovereignty as an obligation toward others. This, in turn, propelled the notion that there are different types of sovereign actors in the international system. Failed states and “rogue states,” for example that are “unwilling or unable” to protect their citizens and the citizens of other states from harm, thus have become legitimate targets for foreign intervention (Albright, 1998; Blair, 1999; Jackson, 2000, p. 360; see also Reinold, 2014; Teitel, 2011).

Third, although international law in substance remains conservative as to the reading of the rules on the use of force, actual practice indicates otherwise. States interpret international law and norms broadly, eventually suggesting “an increasing acceptance of the notion that irresponsible sovereigns lose some of the protections normally afforded to sovereign states” (Reinold, 2011a, p. 396). The relevance of international law has thus by no means diminished. This is not to make any judgments about the substance of international law. Rather, this is to indicate that international law remains a focal point in the discourse on international order as the practice of targeted killing and the use of violence more generally illustrates (e.g. Fisher, 2006; Gunneflo, 2016). In fact, the laws of war are rather permissive than constraining regarding the use of violence by sovereign actors, as a recent study by Hurd (2017a) indicates. In fact, as Hurd (2017a) argues, “international law is a resource which increases state power” (p. 1). In the case of targeted killing this is, for example, obvious in the “lawfare” (Grayson, 2012, pp. 121–122) surrounding this practice, indicating its effects on international law and the use thereof to either justify targeted killing or evade international law by complying with its letter but violating its purpose (Búzás, 2016). After this discussion of the current transformation of targeted killing and its facilitating conditions, the next section of our article summarizes the arguments that the individual contributions to this special issue make regarding the impact that this transformation has on the global international order.

## Targeted killing and international order—contributions to the special issue

**Figure 2.**
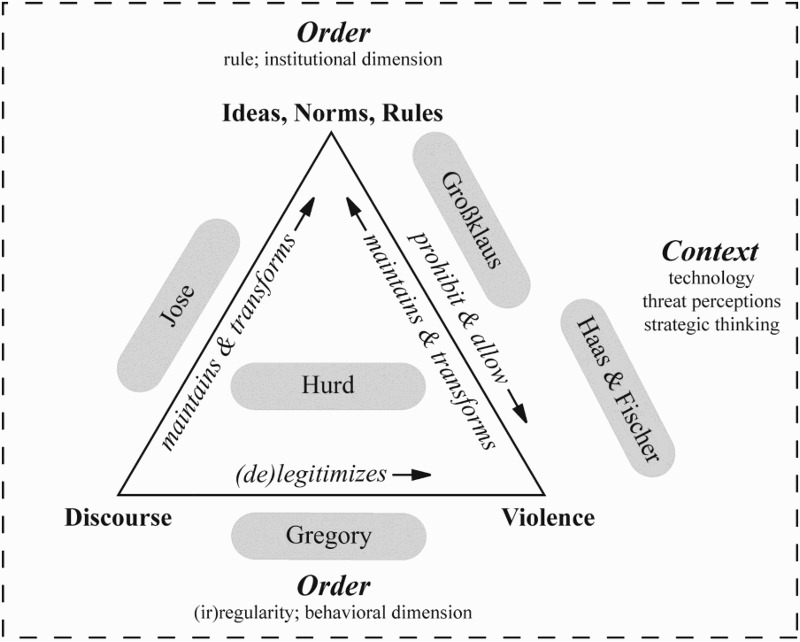
Contributions to the special issue.

In their analysis of whether and how the current transformation of targeted killing is transforming international order, the contributions to our special issue address all three dimensions of our theoretical framework (see Figure 2). In the first article, Gregory (2017) focuses on the discursive process through which actors (re)interpret existing elements of the current international order and legitimize their uses of violence. Gregory analyzes how decision-makers in the United States have (re)interpreted the *jus in bello* principle of noncombatant immunity to legitimize targeted-killing strikes. He argues that this discursive process essentially broadened the range of legitimate targets and blurred the distinction between combatants and noncombatants. In addition, Gregory traces the discursive moves through which U.S. decision-makers constructed the (more or less unlimited) geographical scope of targeted-killing missions.

While Gregory investigates the interpretation of existing principles and norms, Jose (2017) analyzes the contested process of norm emergence. In her article, Jose identifies the United States as the key entrepreneur of a new norm, which renders targeted killing an appropriate form of violence, and Human Rights Watch as its key antagonist or norm suppressor. She finds that Human Rights Watch first attempted to entirely suppress the emergence of this norm and then refocused its efforts on the substance of the norm once it had become clear that the norm may emerge. So Human Rights Watch eventually concentrated on preventing the rise of an overly permissive norm. In her analysis of factors that influence the success of norm advocacy and suppression, Jose addresses the role of the historical context, the productive power of entrepreneurs and suppressors, and how these actors frame their respective messages.

The article by Großklaus (2017) expands the focus from an individual norm to the interaction of principles and norms that make up the current international order. He rejects the view that the attacks of 9/11 and the ensuing war against terrorism triggered the erosion of the anti-assassination norm and argues instead that the norm’s transformation is a long-term process that unfolds within the meta-norms structuring international order. Großklaus identifies the norm of state sovereignty and liberal norms as the normative context for the emergence and transformation of the anti-assassination norm. Both meta-norms have shaped the assassination norm: While sovereignty precludes attacks on certain individuals such as heads or representatives of states, liberal norms result in the prohibition of certain (treacherous, perfidious) ways of killing. Building on this understanding of the anti-assassination norm as an “amalgam,” Großklaus argues that its transformation in large parts follows from the discursive separation between targeted killing and liberal meta-norms.

Haas and Fischer (2017) address the contextual dimension of our theoretical framework and how it will shape the future transformation of targeted killing and international order. Haas and Fischer expect that technological advances, specifically in terms of autonomous weapons systems employed in a context of existing military-theoretical frameworks that emphasize leadership decapitation, will lead to an expansion of targeted-killing missions beyond the realm of counterterrorism operations. In their view, these expanded applications of targeted killings would not only increase the risk of escalating crises at the behavioral level, but would also affect the institutional dimension of the current international order. In other terms, they see a potential for the (further) erosion of the ideas, principles, and norms that govern the use of violence in the global international order.

In the final article, Hurd (2017b) offers a critical appraisal of the special issue and identifies avenues for further research on the nexus between the transformation of targeted killings and international order. Hurd illustrates that the theoretical implications of the contributions of this special issue have far greater consequences than just for the research on targeted killing. Rather, he argues for a new stream of research in favor a conceptual sophistication grounded in empirical evidence.

## Conclusion

This article argued that we are currently witnessing a transformation of targeted killing. The number of states engaging in this from of violence is increasing, as is the overall number of targeted-killing missions. Moreover, the development and proliferation of drone technology constitute a technological revolution in targeted killing. Finally, states are starting to abandon the cloak of secretiveness and denial and to (hesitantly) engage in the public legitimization of their targeted-killing operations. Although this transformation has kindled considerable interest in many dimensions of targeted killing, only few scholars address the question of whether and how its current transformation is at the same time transforming the ideas, norms, and rules that constitute the global international order. A systematic and sustained engagement with this question is highly relevant not only for the academic study of international order and international law, but also for policy-making. As a growing number of states are considering or seeking the acquisition of armed drones and would thus have the technological infrastructure for targeted killings within their reach, political and military decision-makers require a thorough and balanced debate on the broader impact of employing targeted killing.

Our article seeks to encourage this debate and to provide a conceptual foundation for the subsequent contributions to our special issue on “The transformation of targeted killing and international order.” To achieve these goals, we introduced a concept of international order and a theoretical framework that conceives the transformation and maintenance of an order as a dynamic interplay between violence and discursive processes as the behavioral dimension of an international order and the ideas, rules, and norms that make up its institutional dimension. In a next step, we argued for a distinction between a concept of targeted killing as a form of violence and judgments about its legal and moral legitimacy. In line with this distinction, we first offered a consciously broad definition of targeted killing and then introduced a fourfold typology of targeted-killing acts on the basis of their legal and moral legitimacy. After this conceptual groundwork, the article shifted the focus on the current transformation of targeted killing and its facilitating conditions and thus established a point of departure for the subsequent contributions, which we summarized in the context of our theoretical framework in the last section of our article.

As this special issue is intended to encourage the debate on the nexus between targeted killing and international order, we close this introductory article by pointing to avenues for further research. First, future studies should inquire into whether and how argumentative patterns of targeted killing are diffusing among state and non-state actors. In other terms, they should analyze the extent to which actors adopt and adapt legal and moral legitimizations of targeted killings, as well as the interpretations of existing legal and moral principles. Second, scholars should also investigate whether the current discourse about state-sponsored killing of terrorist actors also has an impact on the way state and non-state actors evaluate the legitimacy of other types of targeted killing, such as the killing of state leaders. Haas and Fischer (2017) argue in their contribution to this special issue that technological developments and military theory could facilitate the expansion of targeted killing beyond the context of counterterrorism. A spillover effect from the current debate about the targeted killing of terrorist actors could be a further facilitating condition in this respect. Finally, future studies should move beyond the focus of this special issue by analyzing the impact of targeted killing on regional orders. As we mentioned in an earlier section of this article, scholars have started to analyze the impact of targeted killing on regional conflicts. This agenda should be complemented by a focus on how the use and legitimation of targeted killing affect the ideas, norms, and rules that constitute regional orders.

## Notes

1. Gunneflo’s (2016) recent study of targeted killing by the United States and Israel cuts across the first and second branches as it offers a very detailed account of how these two countries came to adopt targeted killing and a meticulous inquiry into legal aspects.

2. Hayek (1973, p. 37) distinguishes between endogenous and exogenous order. Likewise, the English School of International Relations distinguishes between (evolved) primary institutions and (designed) secondary institutions. See Buzan (2004, 2014) and Flockhart (2016).

3. While Jervis (2012, p. 412) and Bull (1995, p. 10) conceive the existence of a system of actors in terms of their “interconnections and interactions” (Jervis, 2012, p. 412), Gilpin (1981, pp. 26–27) builds on the work of Mundell and Swoboda and identifies “diverse entities,” “regular interactions,” and “forms of control” as constitutive elements of a system. The latter element denotes the existence of an order that shapes the behavior of actors in the system.

4. As Bially-Mattern (2005) notes in this context,
[t]here are many different types of orders in world politics, many of which are not exclusively constituted by the relationships among states and many of which may not directly involve all states. For instance, orders can emerge out of the relationship between states and non-state actors … or through relationships among different types of nonstate actors … . But as the phrase “international order” is used among IR scholars, it refers only to orders that emerge among states. (pp. 28–29). See Ralph (2007) on state and non-state orders.

5. Our perspective on the Westphalian order by and large follows the tradition of the English School of International Relations. See Buzan (2004, 2014) and Flockhart (2016, p. 14).

6. As a number of scholars have highlighted, political practice has frequently violated the idea of sovereignty as autonomy, equality, and noninterference. See, for example, Krasner (2001) and Simpson (2004).

7. C. Thomas (2011) provides a useful discussion and definition of violence as “an intentional act designed to cause harm, which is direct and physical or psychological” (p. 1834). The emphasis on intentionality follows from Thomas’ focus on violence between states and thus excludes nonintentional forms of violence. A number of scholars have identified the management of violence as a key function of political orders (e.g. Bull, 1995; Fukuyama, 2014, p. 583; Kalyvas et al., 2008, p. 1; North et al., 2009, pp. 13–18; Phillips, 2011, pp. 16–17). The development of sovereignty itself was advanced by endemic, religiously motivated violence in Europe. As Johnson (2014) notes in this context, [t]he [Westphalian] conception of sovereignty aimed importantly at preventing wars over rival religious beliefs, but while it succeeded in this aim (though in the last century it has not prevented conflicts inflamed by secular ideologies—a functional equivalent to religion—and by the rise of militant radical Islamism), it also provided a ground for two unseen and undesirable new developments: the initiation of war by governments of their own choosing on the grounds of the defense of national territory and, through the principle of noninterference. (pp. 137–138)

8. See Schmitt (1992) for a detailed discussion. As the Convention notes in its preamble, “crimes against diplomatic agents and other internationally protected persons jeopardizing the safety of these persons create a serious threat to the maintenance of normal international relations which are necessary for the cooperation among states.”

9. See also Dinstein (2011, part II) for a comprehensive treatment of the prohibition of the use of force. Articles 53 and 107 of the UN Charter exclude the use of force against so-called enemy states during World War II (i.e. Germany and Japan) from the prohibition of the use of force.

10. Holzscheiter (2014) distinguishes between two approaches to the study of discourse. The macro-structural approach focuses on broad structures of signification, whereas the micro-structural approach puts the “analytical emphasis on the real-time communicative processes in which agents actively construct, re-negotiate, and transform intersubjectively shared interpretations of reality” (Holzscheiter, 2014, p. 147). Our use of the term “discourse” follows this micro-structural understanding and refers to processes of creating intersubjective meanings.

11. In an exchange over the Caroline incident of 1837, U.S. Secretary of State Daniel Webster noted that “It will be for that Government to show a necessity of self-defence, instant, overwhelming, leaving no choice of means, and no moment for deliberation.” For the expanded interpretation of imminence, see Watson (2017, p. 3).

12. This premise is by no means uncontested. As Carter (2015) notes, a number of scholars “deny that any political concept can be value neutral in this way. The *point* of political concepts like freedom, power, and justice, they say, is to make ethical evaluations” (p. 283).

13. Knoepfler (2010) elaborates on prominence:
By “prominent person,” I mean that someone who is a leader of some sort or is particularly famous and important. This includes political leaders … but also military leaders, religious figures, rich and influential public figures, “big-time crime bosses,” and leadership within political or social movements, such as revolutionary or terrorist organizations. (p. 477)

14. See C.A. Thomas (2014) for this distinction. He defines legal legitimacy and moral legitimacy “as a property of an action, rule, actor, or system which signifies a legal [or moral] obligation to submit to or support that action, rule, actor or system” (p. 735). Thomas (2014) also notes that “[l]egal legitimacy is similar to moral legitimacy in that both assess given objects against a particular normative framework; as such they are both sometimes grouped together as forms of ‘normative legitimacy’” (p. 735).

15. The killing of Admiral Yamamoto, the Commander-in-Chief of the Imperial Japanese Combined Fleet and architect of the attack against Pearl Harbor, by the United States in April 1943 (Operation Vengeance) is a well-known historical example of this type of targeted killing. In contrast to the Yamamoto case, the targeted killing of SS general Reinhard Heydrich, the governor of the German protectorate of Bohemia and Moravia, in May 1942 (Operation Anthropoid) was not legally legitimate, despite Heydrich’s status as an enemy combatant. As Dinstein (2016) explains, “Heydrich—as a military officer—was a lawful target, just like Yamamoto. Still, the act constituted unlawful combatancy, since Heydrich was killed by members of the Free Czechoslovak army (parachuted from London) who were not wearing uniforms” (p. 118).

16. See also Walzer (2013), who argues that:
… even with tyrants, a trial is preferable to an assassination whenever it is possible to bring down the tyrannical regime without killing its leader. In wartime, international law bars the killing of political leaders on the grounds that they are the ones who will in the end negotiate a peace treaty. But some political leaders, with whom one can’t imagine negotiating, are legitimate targets—Hitler the obvious example. Killing Hitler would have been “extra-judicial” but entirely justified. In a similar way, one can argue that the targeted killing of Reinhard Heydrich did not have legal legitimacy due to its treacherous character, but had moral legitimacy in view of Heydrich’s leading role in the Holocaust and his actions in the German protectorate. However, the moral legitimacy of this killing has also been contested given the massive German reprisals that followed Heydrich’s death and were to be expected given his position within the Nazi hierarchy. Hauner (2007) counters this criticism as follows:
those who argue that sparing Heydrich could have saved thousands of lives, must also face the fact that every day of his service to the Third Reich meant the further perfection of the Nazi killing machine and … a more ruthlessly efficient genocide. By killing one of Hitler’s ablest lieutenants, the brutal regime and its collaborators were shaken, and the occupied peoples of Europe given hope. (p. 89)

17. A targeted killing of Saddam Hussein by Coalition Forces during Operation Iraqi Freedom would have been a case of this type of killing. Coalition forces actually targeted Hussein and his close circle during Operation Iraq Freedom, but to no avail. His targeted killing would have been legal because Iraq and the members of the coalition were in an international armed conflict and so international humanitarian law applied, which allows the killing of enemy combatants. Given his position as commander-in-chief of Iraq’s armed forces and his direct involvement in the conduct of military campaigns (Sasson, 2012, pp. 137–139), Hussein was a legal target, despite his position as head of state, which would have afforded him protection under the New York Convention. Yet, if one follows the revisionist view, the moral legitimacy of this killing would have been dubious because the decision to use force against Iraq appears to be on shaky ground in terms of *jus ad bellum* principles*.* In their analysis of the Iraq Intervention on the basis of Just War Theory, Enemark and Michelsen (2005) conclude “that the Coalition’s military campaign was unjust” (p. 562) because it violated all but one (*right intention*) of the *jus ad bellum* principles. If one accepts this judgment, a targeted killing of Saddam Hussein would have been legally legitimate but morally illegitimate. See also Henderson (2009, pp. 110–116), Schmitt (2006, pp. 161–164), and Solis (2016, pp. 587–588) for the debate about the legality of killing state leaders during international armed conflicts.

18. Scholars frequently use the label of *assassination* for at least two of these four types (types II and IV) of targeted killing.

19. This includes the actual possession of drones and their development. See also the reporting on https://www.newamerica.org/in-depth/world-of-drones/.

## Appendix

**Table A1.** Number of targeted-killing strikes (January 2002–November 2016).

Data source	Attacker	Target	2002	2003	2004	2005	2006	2007	2008	2009	2010	2011	2012	2013	2014	2015	2016	Total
BIJ																		
	United States	Pakistan	NA	NA	1	3	2	5	38	54	128	75	50	27	25	13	3	**424**
		Yemen	1	0	0	0	0	0	0	3	33	34	124	33	36	32	51	**347**
		Somalia	NA	NA	NA	NA	NA	5	2	1	0	5	3	2	3	13	15	**49**
		Afghanistan	NA	NA	NA	NA	NA	NA	NA	NA	NA	NA	NA	NA	NA	236	410	**646**
			**1**	**0**	**1**	**3**	**2**	**10**	**40**	**58**	**161**	**114**	**177**	**62**	**64**	**294**	**479**	**1466**
CSTK																		
	United States	Pakistan	NA	NA	1	3	3	5	36	56	136	76	49	27	24	14	3	**433**
		Yemen	1	0	0	0	0	0	0	2	2	20	60	31	29	29	23	**197**
		Somalia	NA	NA	NA	NA	NA	NA	NA	NA	NA	8	2	1	3	7	6	**27**
	Israel	Gaza Strip (Palestine)	NA	NA	10	3	4	0	0	0	3	8	4	22	98	0	0	**152**
		Sudan	NA	NA	NA	NA	NA	NA	NA	3	0	1	0	0	0	0	0	**4**
		Egypt	NA	NA	NA	NA	NA	NA	NA	NA	NA	NA	NA	1	0	0	0	**1**
		Lebanon	NA	NA	NA	NA	NA	NA	NA	NA	NA	NA	NA	1	0	0	0	**1**
	Russia	Qatar	NA	NA	1	0	0	0	0	0	0	0	0	0	0	0	0	**1**
	Colombia	Ecuador	NA	NA	NA	NA	NA	NA	1	0	0	0	0	0	0	0	0	**1**
	Turkey	Syria	NA	NA	NA	NA	NA	NA	NA	NA	NA	NA	NA	NA	1	9	2	**12**
			**1**	**0**	**12**	**6**	**7**	**5**	**37**	**61**	**141**	**113**	**115**	**83**	**155**	**59**	**34**	**829**
LWJ																		
	United States	Pakistan	NA	NA	1	2	3	5	35	53	117	64	46	28	24	11	3	**392**
		Yemen	1	0	0	0	0	0	0	2	4	10	41	26	23	23	31	**161**
			**1**	**0**	**1**	**2**	**3**	**5**	**35**	**55**	**121**	**74**	**87**	**54**	**47**	**34**	**34**	**553**
NAF																		
	United States	Pakistan	NA	NA	1	3	2	4	36	54	122	72	48	26	22	10	3	**403**
		Yemen	1	0	0	0	0	0	0	2	1	12	56	25	17	24	37	**175**
		Somalia	NA	2	1	0	0	5	2	1	0	4	2	2	3	5	13	**40**
			**1**	**2**	**2**	**3**	**2**	**9**	**38**	**57**	**123**	**88**	**106**	**53**	**42**	**39**	**53**	**618**
SATP																		
	United States	Pakistan	NA	NA	NA	1	0	1	19	46	90	59	46	24	19	14	3	322
Morehouse (RF)																		
	Russian Federation	Chechnya	NA	NA	6	7	5	0	0	0	0	0	NA	NA	NA	NA	NA	**18**
					**6**	**7**	**5**	**0**	**0**	**0**	**0**	**0**					**0**	**18**
Morehouse (COL)																		
	Colombia	FARC	NA	NA	1	1	0	2	0	0	1	1	NA	NA	NA	NA	NA	**6**
					**1**	**1**	**0**	**2**	**0**	**0**	**1**	**1**					**0**	**6**

Sources: The Bureau of Investigative Journalism (BIJ), www.thebureauinvestigates.com (data retrieved on 15 November 2016). The Center for the Study of Targeted Killing (CSTK) at the University of Massachusetts Dartmouth, http://www.targetedkilling.org/database/list (data retrieved on 15 November 2016). The Long War Journal (LWJ), http://www.longwarjournal.org/ (data retrieved on 15 November 2016). The New America Foundation (NAF), http://securitydata.newamerica.net (data retrieved on 1 July 2016). South Asia Terrorism Portal (SATP), http://www.satp.org (data retrieved on 15 November 2016). Morehouse (RF) taken from Morehouse (2015). Morehouse (COL) taken from Morehouse (2014). Bold numbers are totals.
